# Hepatitis A, B and C prevalence among transgender women and travestis in five Brazilian capitals between 2019-2021

**DOI:** 10.1590/1980-549720240005.supl.1

**Published:** 2024-08-19

**Authors:** Regina Célia Moreira, Maria Amélia de Sousa Mascena Veras, Carolina Amianti, Daniel Jason McCartney, Vanessa Cristina Martins Silva, Marcilio Figueiredo Lemos, Adriana Parise Compri, Elaine Lopes de Oliveira, Katia Cristina Bassichetto, Andréa Fachel Leal, Daniela Ruva Knauth, Laio Magno, Inês Dourado, Lenice Galan, Paula Andrea Morelli Fonseca, Rita Suely Bacuri de Queiroz, Roberto José Carvalho da Silva, Sandra Araujo, Marcia Eiko Miyachi, Claudio de Sousa Soares, Luciana Mitie Kawai Ahagon, Philippe Mayaud, Sandro Sperandei, Ana Rita Coimbra Motta-Castro

**Affiliations:** I Instituto Adolfo Lutz, Virology Center, Viral Hepatitis Laboratory –São Paulo (SP), Brazil.; II Núcleo de Pesquisa e Direitos Humanos em Saúde da População LGBT+ – São Paulo (SP), Brazil.; III Santa Casa de São Paulo, School of Medical Sciences – São Paulo (SP), Brazil.; IV Universidade Federal de Mato Grosso do Sul – Campo Grande (MS), Brazil.; V London School of Hygiene & Tropical Medicine, Faculty of Infectious & Tropical Diseases, Department of Clinical Research – London, United Kingdom.; VI Universidade Federal do Rio Grande do Sul – Porto Alegre (RS), Brazil.; VII Universidade do Estado da Bahia, Department of Life Sciences – Salvador (BA), Brazil.; VIII Universidade Federal da Bahia, Institute of Public Health – Salvador (BA), Brazil.; IX Fiocruz Amazônia, Instituto Leônidas e Maria Deane – Manaus (AM), Brazil.; X São Paulo State Health Secretariat, STD/AIDS Reference and Training Center – São Paulo (SP), Brazil.; XI Laboratory of the STD/AIDS Reference and Training Center – São Paulo (SP), Brazil.; XII Estern Sydney University, Translation Health Research Institute – Penrityh, Australia.; XIII Fundação Oswaldo Cruz – Campo Grande (MS), Brazil.

**Keywords:** Hepatitis A, Hepatitis B, Hepatitis C, Prevalence, Transgender women

## Abstract

**Objective:**

To estimate the prevalence and factors associated with hepatitis A, B, and C in transgender women and *travestis's* networks, in 5 regions of Brazil.

**Methods:**

This cross-sectional study includedtransgender women and *travestis* in five Brazilian capitals (Campo Grande, Manaus, Porto Alegre, Salvador, and São Paulo), between December/2019 and July/2021. All samples were subjected to detection of serological markers of hepatitis virus A (HAV), B (HBV), and C (HCV) infections through rapid tests and chemiluminescent microparticle immunoassays. Positive samples in the screening tests were submitted to detect HBV DNA and HCV-RNA by real-time PCR and genotyped by Sanger sequencing.

**Results:**

Analysis of 1,317 samples showed network prevalence rates of 69.1%, 25.1%, and 1.5% for HAV, HBV, and HCV exposure, respectively. A high susceptibility rate to HBV infection (35.7%) and low prevalence of vaccine response markers (40%) were also observed. Age greater than 26 years, self-declared black/brown skin color, having only primary education, history of incarceration, and use of a condom in the last sexual intercourse with a casual partner were associated with total anti-HAV. Exposure to HBV was associated with age greater than 26 years, self-declared black/brown, history of being a sex worker, and incarceration. Age > 37 years, history of sexual abuse, and frequent alcohol consumption were associated with hepatitis C infection.

**Conclusion:**

The highest prevalence of HAV in this population was found in the North and Northeast regions, and the prevalence found was higher than that in the general population, suggesting greater vulnerability. The prevalence of HCV infection in our study was similar to that observed in the general population.

## INTRODUCTION

In 2016, the World Health Organization (WHO) proposed the elimination of viral hepatitis by 2030, urging countries to address key challenges such as reducing mortality, lowering incidence rates, improving diagnostic availability, and ensuring widespread access to treatment for the entire population^1,2^.

Recent epidemiological studies focusing on key populations (gays and other men who have sex with men (MSM), transgender individuals, people who use alcohol and other drugs, individuals deprived of liberty, and sex workers) highlight the disproportionate burden of infections such as HIV, hepatitis B (HBV), hepatitis C (HCV), and other sexually transmitted infections (STIs)^3,4^. Among these populations, transgender women and *travestis* are particularly at high risk of acquiring STIs such as HIV, syphilis, and hepatitis B^5,6^.

Leaving home/school early, discrimination and prejudice experienced by many of them, especially the poorest and the black, often lead them to social exclusion and, consequently, to prostitution as a survival strategy. Factors directly related to prostitution, such as risky sexual practices, a high number of sexual partners, consumption of illicit drugs and alcoholic beverages, low educational level, and socioeconomic marginalization, contribute to heightened vulnerability to acquiring STIs among this population^5-7^.

HAV infection is closely linked to hygiene conditions within the population and certain sexual practices. In particular, homeless women have a higher prevalence of HAV infection due to increased exposure to contaminated water and food, which facilitates the transmission of fecal-oral infections^8^.

Vaccines against hepatitis A and B are widely available in Brazil. The hepatitis B vaccine has been administered since the 2000s and has progressively become accessible to adults up to 49 years of age. Initially, the hepatitis A vaccine was limited to individuals with serious conditions such as HIV, immunosuppression, or chronic carriers of hepatitis B and C^8-10^. However, since 2014, it has been included in the National Vaccination Calendar and has been offered to all children from the age of 12 months. Regarding hepatitis C (HCV), the primary strategy involves early diagnosis of active infection, with effective treatment readily available within the public healthcare system, offering a high likelihood of cure for populations at a high risk of exposure to the virus^11-13^.

Based on the information provided, the objective of the present study was to determine the prevalence and factors associated with hepatitis A, B, and C among transgender women and *travestis* across five regions of Brazil, as well as to identify the genotypes or subtypes of hepatitis B virus (HBV) and hepatitis C virus (HCV) in this population.

## METHODS

The current research is a component of the broader project titled “Study on the Prevalence of Syphilis and other Sexually Transmitted Infections (STIs) among *Travestis* and Transgender Women in Brazil: Care and Prevention.” It was conducted as a cross-sectional study across five major Brazilian cities (Campo Grande, Manaus, Porto Alegre, Salvador, and São Paulo), spanning from December 2019 to July 2021.

Participants were recruited using respondent-driven sampling (RDS). Upon signing the informed consent form, all participants were administered a questionnaire covering sociodemographic information, behavioral factors, and history of hepatitis A and B vaccination.

After completing the questionnaire, participants underwent venipuncture for blood collection to perform rapid tests (RT) for anti-HIV-1/2, anti-*Treponema pallidum*, HBV surface antigen (HBsAg), and anti-HCV. All blood samples were then sent to São Paulo, specifically to the Viral Hepatitis Laboratories of Instituto Adolfo Lutz (IAL) and the STI/AIDS Reference and Training Center (*Centro de Referência e Treinamento em IST/Aids* – CRT) for serological and molecular testing. The samples underwent detection of total anti-HAV, anti-HAV IgM, total anti-HBc, and anti-HBs using the chemiluminescent microparticle immunoassay (CMIA) technique, utilizing the Architect^®^ i1000 system (Abbott Ireland, Diagnostics Division, Sligo, Ireland), following the manufacturer’s instructions.

Samples that tested reactive for HBsAg (RT) and anti-HCV underwent detection and quantification of HBV DNA and HCV RNA using real-time PCR (qPCR) (Abbott^®^ Real Time, Des Plaines, USA). Genotyping of the samples with detected viral load was carried out using conventional PCR techniques, following protocols described by Sitnik et al.^14^ and Gomes-Gouvêa et al.^15^ for HBV and, for HCV genotyping, Santos et al.^16^. The amplified genetic materials were sequenced using Sanger method^17^, following the instructions in the package insert for the BigDye™ Terminator v3.1 Cycle Sequencing Kit (Applied Biosystems, Foster City, CA, USA) and analyzed using the ABI3500 Automatic Sequencer (Applied Biosystems, Thermo Fisher Brand, Foster City, CA, USA). Genotypes were confirmed using the Geno2pheno^18^ genotyping tool. Further methodological details of the study can be found in Veras et al.^19^

The study outcomes comprised exposure to and presence of active disease from HAV, HBV, and HCV viruses. For HAV, exposure (HAV-Expo) was identified by total anti-HAV marker positivity, whereas the prevalence of active disease (HAV-Prev) was determined by the presence of the anti-HAV IgM marker. Regarding HBV, exposure (HBV-Expo) was ascertained by positivity for total anti-HBc and/or HBsAg, whereas active disease prevalence (HBV-Prev) was indicated by the presence of HBsAg associated with total anti-HBc. Participants with isolated positivity for anti-HBs (HBV-Vac) were considered immune due to hepatitis B vaccination, whereas those without any positive markers for HBV were classified as HBV “susceptible” to HBV infection (HBV-Susc). HCV exposure (HCV-Expo) was detected by anti-HCV positivity, whereas active disease prevalence (HCV-Prev) was determined by investigating the presence of HCV RNA viral load. The study included a total of 20 exposure variables potentially associated with each of the six outcomes mentioned above, based on the existing literature.

The main outcomes were presented as absolute and relative frequencies, along with a 95% confidence interval for the total sample and city/collection site. The exposure variables were also depicted in absolute and relative frequencies. Univariate logistic models were employed for the exploratory analysis of associations with each outcome. The selection of the final multivariate models began by identifying variables with a bivariate model p-value of 0.3^20^ or less as candidates for inclusion. The GLMERSelect function from the “StatisticalModels” package in R, version 4.2^21^, was used for stepwise selection of fixed effects and interactions in a generalized linear (logistic) mixed-effects model, with city/collection site as random intercepts. The results of the associations were presented as point odds ratios (OR) with their respective 95% confidence intervals.

The project was approved by the Research Ethics Committee of the Santa Casa de Misericórdia de São Paulo (CAAE 05585518.7.0000.5479; opinion n°: 3.126.815 – 30/01/2019), as well as by other participating institutions.

## RESULTS

A total of 1,317 transgender women and *travestis* participated in the study, with 403 recruited in São Paulo, 192 in Porto Alegre, 201 in Salvador, 181 in Campo Grande, and 340 in Manaus. Across all five capitals, the mean age was 32.5 years (SD 10.0), with a median age of 31 years. The majority identified as transsexual women (66.8%), were single (70.1%), and reported completing or having incomplete high school education (54.3%). Regarding occupation, 24.6% reported being self-employed, 21.3% engaged in sex work, and 22.5% were unemployed at the time of the interview.

Tables 1 and 2 show the prevalence of serological markers of HAV, HBV, and HCV in the study population. The final number of samples analyzed varied according to each hepatitis and was lower than the number of samples collected due to the insufficient volume of serum/plasma/whole blood to carry out serological and/or molecular tests. A total of 1,270, 1,206, and 1,285 samples were analyzed to investigate serological markers of infections caused by HAV, HBV, and HCV, respectively.

**Table 1:** Prevalence of serological markers of hepatitis A, B, and C among transgender women and *travestis*.

Serological markers	n	Positive	%	IC95%
Hepatitis A				
Total anti-HAV	1,270	877	69.1	66.6–71.6
Anti-HAV IgM	1,270	34	2.7	1.8–3.6
Hepatitis B				
Exposure to HBV (anti-HBc and(or) HBsAg)	1,206	294	24.4	22.0–26.8
Isolated anti-HBs	1,206	482	40.0	37.2–42.8
Absence of exposure marker	1,206	430	35.7	33.0–38.4
Hepatitis C				
Anti-HCV	1,285	19	1.5	0.8–2.2

CI: confidence interval, HBV: hepatitis B virus; HBsAg: HBV surface antigen; anti-HBs: antibody against the HBV surface antigen; total anti-HBc: antibody against the HBV core antigen; anti-HAV IgM: IgM class antibodies against the hepatitis A virus; total anti-HAV: total antibodies against the hepatitis A virus; anti-HCV: total antibodies against the hepatitis C virus.

**Table 2:** Prevalence of serological markers of hepatitis A, B, and C among transgender women and *travestis* by Brazilian capital.

	HAV_Total	HAV_IgM	Anti_HBc T	HBsAg	HBV_Susc	Anti-HBs	HCV_Expo
São Paulo							
P (95%CI)	71.3 (66.9–75.7)	2.7 (0.9–4.5)	28.3 (24.0–33.0)	1.8 (0.2–3.4)	23.8 (19.6–28.0)	47.8 (42.9–52.7)	0.7 (0–2.5)
n/N	286/401	11/401	114/400	7/400	95/400	191/400	3/402
Porto Alegre							
P (95%CI)	57.4 (50.3–64.5)	1.6 (0–5.5)	37.1 (30.0–44.2)	2.7 (0–6.0)	26.3 (19.8–32.8)	36.6 (29.5–43.7)	5.3 (1.6–9.0)
n/N	108/188	3/188	69/186	5/186	49/186	68/186	10/188
Salvador							
P (95%CI)	58.8 (51.5–66.1)	2.8 (0–6.2)	25.0 (17.1–32.9)	0 (---)	38.7 (29.9–47.5)	36.3 (27.6–45.0)	0 (---)
n/N	104/177	5/177	31/124	0/124	48/124	45/124	0/182
Manaus							
P (95%CI)	80.3 (76.0–84.6)	3 (0.9–5.1)	14.8 (10.9–18.7)	1.8 (0–3.7)	48.4 (43.0–53.8)	36.8 (31.6–42.0)	1.2 (0–3.1)
n/N	269/335	10/335	50/337	6/337	163/337	124/337	4/336
Campo Grande							
P (95%CI)	65.1 (57.8–72.4)	3 (0–6.6)	18.9 (12.5–25.3)	1.3 (0–12.7)	47.2 (39.3–55.1)	34 (26.5–41.5)	1.1 (0–11.1)
n/N	110/169	5/169	30/159	2/159	75/159	54/159	2/177

P: positivity; CI: confidence interval; n: total positive results; N: total analyzed; Total HAV: total antibodies against hepatitis A virus; HAV IgM: IgM class antibodies against the hepatitis A virus; Anti-HBc T: total antibodies against the hepatitis B virus core antigen; HBsAg: hepatitis B virus surface antigen; HBV_Susc: absence of serological marker for hepatitis B virus; Anti-HBs: antibody against the surface antigen of the hepatitis B virus; HCV_Expo: total antibodies against the hepatitis C virus.

Regarding markers for HAV, 877 participants (69.1%, 95%CI: 66.6–71.6) tested positive for total anti-HAV. The highest prevalence was observed in Manaus (80.3%), followed by São Paulo (71.3%), Campo Grande (65.1%), Salvador (58.8%), and Porto Alegre (57.4%). Anti-HAV IgM positivity was observed in 34 samples (2.7%, 95%CI: 1.8–3.6). The prevalence of active disease varied between 3% in Manaus and Campo Grande, 2.8% in Salvador, 2.7% in São Paulo, and 1.6% in Porto Alegre.

A total of 1,206 samples were tested for serological markers of HBV exposure. Among them, 294 tested positive for total anti-HBc and/or HBsAg, resulting in a prevalence of 24.4% (95%CI: 22.0–26.8). São Paulo (28.3%) and Porto Alegre (37.1%) had the highest rates of HBV exposure. HBsAg associated with total anti-HBc was detected in 22 participants, with only one showing isolated HBsAg. The presence of HBsAg in RT was observed in 1.8% of the samples (22/1,206; 95%CI: 1.2–2.7), ranging from 2.7% in Porto Alegre to 1.3% in Campo Grande. Total anti-HBc associated with anti-HBs was detected in 24.4% of the samples, and isolated anti-HBc was found in 3.4% of the samples.

Isolated positivity for the anti-HBs marker was observed in 40.0% of the participants (482/1,206), indicating a vaccine immune response to HBV. The highest rate was observed in São Paulo (47.8%), followed by Manaus (36.8%), Porto Alegre (36.6%), Salvador (36.3%), and Campo Grande (34.0%). Conversely, no serological marker for hepatitis B was detected in 35.7% of the samples (430/1, 206), suggesting susceptibility to HBV infection. The highest susceptibility rate to HBV infection was found in Manaus (48.4%), followed by Campo Grande (47.2%), Salvador (38.7%), Porto Alegre (26.3%), and São Paulo (23.8%).

Among the 22 samples that tested positive for HBsAg in RT, 19 underwent HBV DNA detection. HBV DNA was detected in 10 samples, and genotyping was performed on three of them. The genotypes identified were HBV-F (02/03) and HBV-A (01/03) (data not shown).

Serological markers of HCV exposure (anti-HCV) were detected in 1,285 blood samples. Among these, 19 samples tested positive for anti-HCV, resulting in a prevalence of 1.5% (95%CI 0.8–2.2). The prevalence rate of HCV exposure varied from 5.3% in Porto Alegre to 0.7% in São Paulo. No positive samples for anti-HCV were found in Salvador. Manaus and Campo Grande had anti-HCV antibody prevalence rates of 1.2% and 1.1%, respectively (Tables 1 and 2).

Nineteen samples positive for anti-HCV antibodies in the TR were sent to the IAL hepatitis laboratory for HCV RNA research. In three samples, it was not possible to perform the assay due to insufficient sample volume, and eight samples presented undetectable results. HCV RNA was detected in eight samples and was genotyped.

The most prevalent genotype was 3a, identified in 50.0% (04/08) of the samples, followed by genotype 1a, accounting for 25.0% (02/08), and genotype 1b, which also represented 25.0% (02/08) of the samples (data not shown).

The results of bivariate and multivariate analyses of factors associated with HAV, HBV, and HCV infections in the study population are presented in Supplementary Tables 1, 2, and 3, respectively. After multivariate analysis, for total anti-HAV positivity, the following variables remained associated: age over 26 years, self-identification as black/brown, only basic education, history of incarceration, and condom use in the last sexual intercourse with a casual partner. Protective factors included not having had sexual intercourse in the last six months and being homosexual, gay, or lesbian. Regarding HBV infection, the following variables were associated with the presence of serological markers after multivariate analysis: age over 26 years, self-identification as black/brown, involvement in sex work, being in a relationship, unstable housing (homeless or without a fixed address, shelter or institution, boarding house/hostel, or house of prostitution), and history of incarceration. However, history of sexual abuse was found to be a protective factor against HBV infection. For HCV infection, factors associated after multivariate analysis included age equal to or greater than 37 years, history of sexual abuse, and alcohol consumption four or more times a week.

## DISCUSSION

The findings of the current study suggest that vaccination coverage against hepatitis A and B among the transgender women and *travestis* participants was low, despite the availability of these vaccines in the Brazilian Unified Health System (*Sistema Único de Saúde* – SUS)^22^. This trend is consistent with previous research conducted in San Francisco, USA, by Choi et al.^23^, as well as studies conducted in Brazil by Rezende^24^ and Oliveira et al.^25^. These results underscore the vulnerability of the transgender women and *travestis* population to HBV infection and emphasize the importance of implementing strategies to raise awareness and enhance participation in vaccination programs.

The highest prevalence of confirmed hepatitis A in Brazil is concentrated in the North and Northeast regions, accounting for 55.4% of all cases, with the state of Amazonas exhibiting the highest prevalence of 8.35%^26^. Similarly, this study’s findings reflect this pattern, with Manaus showing the highest prevalence among the five participating cities, followed by São Paulo. The elevated prevalence observed in São Paulo may be attributed to a significant increase in hepatitis A cases during an outbreak among MSM and gay men within the same age group^25^ studied. This epidemic, occurring between 2016-2017, resulted in 155 hospitalizations, including four cases of fulminant hepatitis, two of which were fatal^27^.

A notably higher prevalence of total anti-HAV among black/brown transgender women is evident, which aligns with previous findings associating this demographic variable with the presence of an HAV infection marker^26^. It is crucial to highlight that black transgender/*travestis* individuals tend to initiate sexual activity earlier than their white counterparts and report higher incidences of sexual violence during childhood and adolescence, as well as experiences of homelessness, factors that increase their exposure and susceptibility to fecal-oral infections^27,28^. These findings are consistent with data from previous studies, such as that by Castro et al.^29^, who observed a prevalence of 69.7% among transgender women in the Central-West region of Brazil. Given that the population in this study was over 18 years old and, as already described in the literature^30^, cases of acute infection in adults may present with more severe clinical manifestations. Vaccination against hepatitis A in the transgender women and *travestis* population and the promotion of safe sex practices, including the use of condoms during oral-anal sex, are essential preventive measures to mitigate the risk of hepatitis A transmission.

Additionally, there is a finding that contradicts plausibility and the literature^8,27^. Exposure to sexual violence appeared to be a protective factor in the present study, similar to the association of hepatitis A with the use of condoms in the last relationship. These associations may be confounded by unmeasured factors.

With regard to hepatitis B, the global prevalence of markers of exposure to HBV found in this study, in the transgender women and *travestis* studied, was higher than that found in the population-based survey, carried out between 2005 and 2009, in the 26 capitals and the Federal District (7.4%; 95%CI 6.8–8.0), suggesting greater vulnerability of this population to HBV infection^31^. However, it was similar to that found in Central-West Brazil in 2018^24^ and lower than the prevalence found in other Latin American countries such as Argentina^32^ (40.2 %) and Uruguay^6^ (50.5 %).

The HBsAg positivity observed in the transgender women and *travestis* studied was higher than the prevalence of HBsAg found in the population-based study carried out in Brazilian capitals^30^. It was also similar to that found in Argentina (1.9%)32 and in Campo Grande-MS (2.7%)^24^ in transgender people and sex workers. However, it was lower than the rates found in countries such as Pakistan^33^ (3.4%) and Uruguay^6^ (3.0%).

Multivariate analysis revealed that age > 26 years was independently associated with HBV infection. This association is likely due to the increased risk of viral exposure over the course of one’s lifetime, particularly through sexual contact, a pattern consistent with previous research findings^34,35^. Additionally, engagement in sex work, either currently or in the past, has emerged as another factor linked to hepatitis B infection in this population, as has been observed in other studies^35-37^. Notably, a significant proportion of the studied population reported either current or past involvement in sex work, a trend consistent with prior studies^7,24,38^. Research has indicated that factors such as financial needs, social stigma, limited employment opportunities, and marginalization in healthcare contribute to transgender individuals’ engagement in sex work^39,40^. Such circumstances predispose them to increased risk of contracting STIs, including HBV, attributed to factors like having multiple sexual partners and engaging in risky sexual behaviors^34,38,41^.

Socioeconomic factors, including unstable housing conditions, have been identified as significant contributors to HBV exposure. Previous research conducted in Brazil^7,24,38^ and India^41^ has also highlighted the low socioeconomic status prevalent among transgender women. This socioeconomic status often correlates with precarious housing conditions characterized by inadequate hygiene, sanitation, and increased exposure to violence, all of which elevate the risk of infection transmission.

Hepatitis C is important in the population of transgender women, mainly due to transmission through the use of injectable and/or inhalable drugs; people deprived of liberty; and people infected with HIV^12^. The highest detection rate of HCV was found in the capital of Rio Grande do Sul, the state with the highest rate of infection in the country^26^. However, overall, the prevalence of HCV in the study population was similar to that found in the general population, which, according to the national survey, was 1.38%^31^.

The prevalence of HCV within the studied group contrasts notably with findings from the USA. Two RDS studies conducted with transgender women in California in 2017 and 2020 reported prevalence rate of 23.8% and 23.9%, respectively^42,43^. Additionally, the association of age as a risk factor for HCV, already described in the literature^32^, appears in both Brazilian and American contexts.

The association between HCV infection and alcohol consumption, as well as a history of sexual abuse, may stem from broader repercussions of violence against women or children. Such experiences often result in various psychological and behavioral impacts on the victim, increasing the likelihood of engaging in alcohol and/or drug use in the future^44,45^.

This study has several limitations. Given its cross-sectional nature, establishing a causal relationship between the analyzed exposure variables and outcomes is not feasible. Additionally, susceptibility rates and the presence of markers indicating a response to hepatitis B vaccination may have been underestimated, as the levels of anti-HBs antibodies diminish over time and can become undetectable. Memory bias and reliance on self-reporting also represent notable limitations. Moreover, the financial incentives associated with the recruitment method might have biased the participation of predominantly low-income transgender women and *travestis* in the study.

The WHO acknowledges that certain key populations, including transgender individuals, exhibit higher prevalence rates of viral hepatitis compared to the general population. Understanding the epidemiological and molecular aspects of hepatitis A, B, and C within these groups, such as transgender women and *travestis*, is crucial for informing public health policies tailored to their needs^1^. The data presented underscores the significant impact of viral hepatitis on this population, characterized by heightened vulnerability due to factors such as inconsistent adoption of safe sexual practices, history of STIs, social stigma, violence, and barriers to accessing healthcare services. These findings emphasize the importance of targeted public policies aimed at this key population, with a focus on enhancing vaccination coverage, and facilitating access to testing and treatment services.
