# Supplementary material for: Hepatitis A, B and C prevalence among transgender women and travestis in five Brazilian capitals between 2019-2021
Source: Rev Bras Epidemiol. 2024 Aug 19;27(Suppl 1):e240005.supl.1. doi: 10.1590/1980-549720240005.supl.1 (PMC11338541; doi:10.1590/1980-549720240005.supl.1)
Supplement: Supplementary file 1 [file 1980-5497-rbepid-27-suppl1-e240005-s1.docx]

**Tabela suplementar 1**: Características sociodemográficas, clínicas e comportamentais de mulheres trans, 2019-2021, Brasil.

| **Características** | **Total** | | **Anti-HAV**  **Total Positivo** | |  |  | **Odds ratio (IC 95%)** |
| --- | --- | --- | --- | --- | --- | --- | --- |
|  | **N** | **%** | **N** | **%** | **P valor** | **IC 95%** |  |
| ***Idade (anos)*** |  |  |  |  |  |  |  |
| ≤ 25 | 368 | 29,0 | 155 | 17,7 | - | Referência |  |
| 26 - 36 | 507 | 39,9 | 367 | 41,8 | <0,001 | 4,28 (3,16 – 5,80) | 4,00 (2,79 – 5,74) |
| ≥ 37 | 395 | 31,1 | 355 | 40,5 | <0,001 | 15,09 (10,05 – 22,67) | 14,68 (8,99 – 23,96) |
| ***Etnia*** |  |  |  |  |  |  |  |
| Branca | 325 | 25,6 | 189 | 21,6 | - | Referência |  |
| Preta/Parda | 892 | 70,2 | 655 | 74,7 | <0,001 | 1,93 (1,46 – 2,54) | 2,15 (1,50 – 3,08) |
| Outro | 43 | 3,4 | 28 | 3,2 | 0,740 | 1,12 (0,57 – 2,22) | 1,26 (0,53 – 2,99) |
| ***Estado civil*** |  |  |  |  |  |  |  |
| Solteira/separada/viúva | 910 | 71,7 | 617 | 70,4 | - | Referência |  |
| Em um relacionamento | 357 | 28,1 | 257 | 29,3 | 0,102 | 1,26 (0,96 – 1,66) |  |
| ***Escolaridade*** |  |  |  |  |  |  |  |
| Ensino superior | 63 | 5,0 | 38 | 4,3 | - | Referência |  |
| Ensino médio | 890 | 70,1 | 579 | 66,0 | 0,327 | 1,31 (0,77 – 2,23) | 1,48 (0,74 – 2,99) |
| Educação básica | 314 | 24,7 | 259 | 29,5 | <0,001 | 3,50 (1,93 – 6,365) | 2,73 (1,24 – 5,98) |
| ***Renda mensal (sm)**** |  |  |  |  |  |  |  |
| 3 ou mais salários mínimos | 75 | 5,9 | 46 | 5,2 | - | Referência |  |
| 1 ou 2 salários mínimos | 510 | 40,2 | 362 | 41,3 | 0,174 | 1,43 (0,86 – 2,38) |  |
| Menos de 1 salário mínimo | 555 | 43,7 | 379 | 43,2 | 0,455 | 1,22 (0,73 – 2,03) |  |
| ***Profissional do sexo*** |  |  |  |  |  |  |  |
| Nunca | 334 | 26,3 | 187 | 21,3 | - | Referência |  |
| Já trabalhou | 404 | 31,8 | 311 | 35,5 | <0,001 | 2,42 (1,75 – 3,34) |  |
| Sim (parcialmente) | 264 | 20,8 | 193 | 22,0 | <0,001 | 2,43 (1,70 – 3,48) |  |
| Sim (atividade principal) | 264 | 20,8 | 182 | 20,8 | <0,001 | 1,93 (1,36 – 2,73) |  |
| ***Histórico de uso de drogas***  ***(injetáveis e não injetáveis)*** |  |  |  |  |  |  |  |
| Não | 455 | 35,8 | 342 | 39,0 | - | Referência |  |
| Sim | 815 | 64,2 | 535 | 61,0 | 0,059 | 0,77 (0,58 – 1,01) |  |
| ***Histórico de encarceramento*** |  |  |  |  |  |  |  |
| Não | 973 | 76,6 | 630 | 71,8 | - | Referência |  |
| Sim | 290 | 22,8 | 242 | 27,6 | <0,001 | 2,73 (1,95 – 3,84) | 2,09 (1,34 – 3,27) |
| ***Histórico de abuso sexual*** |  |  |  |  |  |  |  |
| Não | 631 | 49,7 | 445 | 50,7 | - | Referência |  |
| Sim | 633 | 49,8 | 426 | 48,6 | 0,305 | 0,88 (0,70 – 1,12) |  |
| ***Uso de preservativo na última relação com parceiro casual*** |  |  |  |  |  |  |  |
| Não | 209 | 16,5 | 122 | 13,9 | - | Referência |  |
| Sim | 334 | 26,3 | 225 | 25,7 | 0,016 | 1,56 (1,09 – 2,25) | 1,99 (1,25 – 3,18) |
| Não teve parceiro casual nos últimos 6 meses | 703 | 55,4 | 512 | 58,4 | 0,001 | 1,77 (1,28 – 2,45) | 1,93 (1,28 – 2,92) |
| ***Nos últimos 6 meses foi diagnosticada com alguma IST*** |  |  |  |  |  |  |  |
| Não | 1038 | 81,1 | 706 | 80,5 | - | Referência |  |
| Sim | 240 | 18,9 | 171 | 19,5 | 0,205 | 1,23 (0,90 – 1,68) |  |
| ***Orientação Sexual*** |  |  |  |  |  |  |  |
| Heterossexual | 996 | 68,7 | 726 | 82,8 | - | - |  |
| Homossexual | 94 | 8,1 | 62 | 7,1 | 0,206 | 0,74 (0,46-1,10) | 0,53 (0,29-0,93) |
| Bissexual | 84 | 7,4 | 55 | 6,3 | 0,260 | 0,76 (0,47-1,22) | 1,04 (0,55-1,96) |
| Panssexual | 80 | 13,7 | 26 | 3,0 | 0,001 | 0,21 (0,13-0,30) | 0,37 (0,19-0,69) |
| Outra | 7 | 1,0 | 3 | 0,3 | 0,115 | 0,30 (0,06-1,30) | 0,24 (0,04-1,29) |

IC: intervalo de confiança; Anti-HAV total: anticorpos totais contra o vírus da hepatite A; IST: Infecção sexualmente transmissível; SM: salário mínimo.

*Renda mensal (sm): R$1.100,00.

**Tabela suplementar 2:** Características sociodemográficas, clínicas e comportamentais de mulheres trans, 2019-2021, Brasil.

| **Características** | **Total** | | **Infecção ativa/crônica pelo HBV** | |  |  | **Odds ratio (IC 95%)** |
| --- | --- | --- | --- | --- | --- | --- | --- |
|  | **N** | **%** | **N** | **%** | **P valor** | **IC 95%** |  |
| ***Idade (anos)*** |  |  |  |  |  |  |  |
| ≤ 25 | 340 | 28,2 | 12 | 4,1 | - | Referência |  |
| 26 - 36 | 483 | 40,0 | 81 | 27,6 | <0.001 | 5,54 (2,96 – 10,39) | 4,37 (2,22 – 8,58) |
| ≥ 37 | 383 | 31,8 | 201 | 68,4 | <0.001 | 32,14 (17,30 – 59,73) | 26,49 (13,44 – 52,21) |
| ***Etnia*** |  |  |  |  |  |  |  |
| Branca | 312 | 25,9 | 67 | 22,8 | - | Referência |  |
| Preta/Parda | 840 | 69,7 | 216 | 73,5 | 0,014 | 1,51 (1,09 – 2,09) | 1,64 (1,10 – 2,46) |
| Outro | 43 | 3,6 | 10 | 3,4 | 0,346 | 1,45 (0,67 – 3,16) | 1,88 (0,69 – 5,10) |
| ***Estado civil*** |  |  |  |  |  |  |  |
| Solteira/separada/viúva | 870 | 72,1 | 218 | 74,1 | - | Referência |  |
| Em um relacionamento | 165 | 13,9 | 41 | 13,9 | 0,68 | 0,92 (0,62 – 1,30) | 1,16 (1,09-2,46) |
| Casada | 168 | 13,7 | 35 | 11,9 | 0,11 | 0,72 (0,48-1,00) | 0,54 (0,32-0,91) |
| ***Escolaridade*** |  |  |  |  |  |  |  |
| Ensino superior | 64 | 5,3 | 14 | 4,8 | - | Referência |  |
| Ensino médio | 846 | 70,1 | 167 | 56,8 | 0,709 | 0,89 (0,48 – 1,66) |  |
| Educação básica | 294 | 24,4 | 113 | 38,4 | 0,012 | 2,3 (1,20 – 4,39) |  |
| ***Renda mensal (sm)**** |  |  |  |  |  |  |  |
| 3 ou mais salários mínimos | 68 | 5,6 | 18 | 6,1 | - | Referência |  |
| 1 ou 2 salários mínimos | 484 | 40,1 | 116 | 39,5 | 0,618 | 0,86 (0,48 – 1,55) |  |
| Menos de 1 salário mínimo | 532 | 44,1 | 138 | 46,9 | 0,999 | 1,00 (0,56 – 1,80) |  |
| ***Moradia*** |  |  |  |  |  |  |  |
| Própria | 306 | 25,4 | 99 | 33,7 | - | Referência |  |
| Alugada | 430 | 35,7 | 107 | 36,4 | 0,036 | 0,7 (0,51 – 0,98) | 0,93 (0,61 – 1,41) |
| Amigos ou família | 329 | 27,3 | 44 | 15,0 | <0,001 | 0,37 (0,25 – 0,56) | 0,60 (0,36 – 1,01) |
| Instável* | 95 | 7,9 | 35 | 11,9 | 0,335 | 1,27 (0,78 – 2,08) | 2,34 (1,24 – 4,40) |
| Outra | 46 | 3,8 | 9 | 3,1 | 0,212 | 0,61 (0,28 – 1,33) | 0,69 (0,27 – 1,76) |
| ***Profissional do sexo*** |  |  |  |  |  |  |  |
| Nunca | 320 | 26,5 | 46 | 15,6 | - | Referência |  |
| Já trabalhou | 391 | 32,4 | 101 | 34,4 | <0.001 | 2,22 (1,49 – 3,28) | 2,76 (1,66 – 4,58) |
| Sim (parcialmente) | 256 | 21,2 | 89 | 30,3 | <0.001 | 2,95 (1,96 – 4,45) | 3,46 (2,03 – 5,91) |
| Sim (atividade principal) | 235 | 19,5 | 58 | 19,7 | 0,004 | 1,89 (1,22 – 2,93) | 2,09 (1,16 – 3,74) |
| ***Histórico de uso de drogas (injetáveis e não injetáveis)*** |  |  |  |  |  |  |  |
| Não | 438 | 36,3 | 94 | 32,0 | - | Referência |  |
| Sim | 768 | 63,7 | 200 | 68,0 | 0,926 | 1,01 (0,75 – 1,37) |  |
| ***Histórico de encarceramento*** |  |  |  |  |  |  |  |
| Não | 921 | 76,4 | 193 | 65,6 | - | Referência |  |
| Sim | 279 | 23,1 | 100 | 34,0 | <0.001 | 2,30 (1,70 – 3,11) | 1,75 (1,19 – 2,57) |
| ***Histórico de abuso sexual*** |  |  |  |  |  |  |  |
| Não | 594 | 49,3 | 155 | 52,7 | - | Referência |  |
| Sim | 605 | 50,2 | 138 | 46,9 | 0,098 | 0,80 (0,61 – 1,04) | 0,64 (0,45 – 0,91) |
| ***Uso de preservativo na última relação com parceiro casual*** |  |  |  |  |  |  |  |
| Não | 193 | 16,0 | 51 | 17,3 | - | Referência |  |
| Sim | 315 | 26,1 | 87 | 29,6 | 0,890 | 1,03 (0,68 – 1,55) |  |
| Não teve parceiro casual nos últimos 6 meses | 675 | 56,0 | 152 | 51,7 | 0,522 | 0,89 (0,61 – 1,29) |  |
| ***Nos últimos 6 meses foi diagnosticada com alguma IST*** |  |  |  |  |  |  |  |
| Não | 976 | 80,9 | 235 | 79,9 | - | Referência |  |
| Sim | 230 | 19,1 | 59 | 20,1 | 0,875 | 0,97 (0,70 – 1,36) |  |
| **Uso de PEP** |  |  |  |  |  |  |  |
| Não | 507 | 42,0 | 144 | 49,0 | - | Referência |  |
| Sim | 127 | 10,5 | 23 | 7,8 | 0,011 | 0,52 (0,32 – 0,86) |  |
| Desconhece | 535 | 44,4 | 114 | 38,8 | 0,308 | 0,85 (0,62 – 1,16) |  |

IC: intervalo de confiança; HBV: vírus da hepatite B; IST: Infecção sexualmente transmissível; SM: salário mínimo; PEP: profilaxia pós exposição.

*Renda mensal (sm): R$1.100,00.

**Moradia instável: em situação de rua ou sem endereço fixo, abrigo ou instituição, pensão/albergue ou casa de prostituição.

**Tabela suplementar 3:** Características sociodemográficas, clínicas e comportamentais de mulheres trans, 2019-2021, Brasil:

| **Características** | **Total** | | **Anti-HCV Positivo** | |  |  | **Odds ratio (IC 95%)** |
| --- | --- | --- | --- | --- | --- | --- | --- |
|  | **N** | **%** | **N** | **%** | **p** | **IC 95%** |  |
| ***Idade (anos)*** |  |  |  |  |  |  |  |
| 18-25 | 373 | 29,0 | 2 | 10,5 | - | Referência |  |
| 26-36 | 513 | 39,9 | 0 | 0 | 0,843 | 0 (0,00 – 9,44) | - |
| ≥ 37 | 399 | 31,1 | 17 | 89,5 | 0,004 | 8,44 (1,99 – 35,72) | 9,54 (2,16 – 42,10) |
| ***Etnia*** |  |  |  |  |  |  |  |
| Branca | 331 | 25,8 | 6 | 31,6 | - | Referência |  |
| Preta/Parda | 899 | 70,0 | 12 | 63,2 | 0,893 | 1,07 (0,39 – 2,94) |  |
| Outro | 44 | 3,4 | 1 | 5,3 | 0,567 | 1,86 (0,22 – 15,69) |  |
| ***Estado civil*** |  |  |  |  |  |  |  |
| Solteira/separada/ viúva | 926 | 72,1 | 18 | 94,7 | - | Referência |  |
| Em um relacionamento | 173 | 13,5 | 1 | 5,3 | 0,180 | 0,26 (0,04 – 1,87) | 0,36 (0,04 – 3,08) |
| Casada | 183 | 14,2 | 0 | 0 | 0,903 | 0,00 (0,00 – 1,24) | - |
| ***Escolaridade*** |  |  |  |  |  |  |  |
| Ensino superior | 65 | 5,1 | 3 | 15,8 | - | Referência |  |
| Ensino médio | 899 | 70,0 | 9 | 47,4 | 0,024 | 0,22 (0,06 – 0,82) |  |
| Educação básica | 318 | 24,7 | 7 | 36,8 | 0,291 | 0,48 (0,12 – 1,88) |  |
| ***Renda mensal (sm)**** |  |  |  |  |  |  |  |
| 3 ou mais salários mínimos | 79 | 6,1 | 1 | 5,3 | - | Referência |  |
| 1 ou 2 salários mínimos | 516 | 40,2 | 8 | 42,1 | 0,797 | 1,31 (0,17 – 10,15) |  |
| Menos de 1 salário mínimo | 558 | 43,4 | 10 | 52,6 | 0,620 | 1,67 (0,22 – 12,83) |  |
| ***Profissional do sexo*** |  |  |  |  |  |  |  |
| Nunca | 338 | 26,3 | 3 | 15,8 | - | Referência |  |
| Já trabalhou | 406 | 31,6 | 7 | 36,8 | 0,227 | 2,28 (0,60 – 8,71) |  |
| Sim (parcialmente) | 267 | 20,8 | 6 | 31,6 | 0,253 | 2,22 (0,56 – 8,77) |  |
| Sim (atividade principal) | 270 | 21,0 | 3 | 15,8 | 0,718 | 1,34 (0,27 – 6,45) |  |
| ***Histórico de uso de drogas (injetáveis e não injetáveis)*** |  |  |  |  |  |  |  |
| Não | 461 | 35,9 | 4 | 21,1 | - | Referência |  |
| Sim | 824 | 64,1 | 15 | 78,9 | 0,406 | 1,63 (0,51 – 5,21) |  |
| ***Consumo de álcool*** |  |  |  |  |  |  |  |
| Nunca | 445 | 34,6 | 6 | 31,6 | - | Referência |  |
| Mensalmente ou menos | 255 | 19,8 | 1 | 5,3 | 0,183 | 0,24 (0,03 – 1,95) | - |
| De 2 a 4 vezes por mês | 328 | 25,5 | 7 | 36,8 | 0,638 | 1,31 (0,42 – 4,05) | 2,00 (0,64 – 6,31) |
| De 2 a 4 vezes por semana | 178 | 13,9 | 2 | 10,5 | 0,710 | 0,74 (0,15 – 3,65) | 0,97 (0,19 – 5,08) |
| 4 ou mais vezes na semana | 73 | 5,7 | 3 | 15,8 | 0,193 | 2,62 (0,62 – 11,13) | 6,09 (1,22 – 30,40) |
| ***Histórico de encarceramento*** |  |  |  |  |  |  |  |
| Não | 985 | 76,7 | 15 | 78,9 | - | Referência |  |
| Sim | 293 | 22,8 | 4 | 21,1 | 0,900 | 0,93 (0,32 – 2,77) |  |
| ***Histórico de abuso sexual*** |  |  |  |  |  |  |  |
| Não | 634 | 49,3 | 6 | 31,6 | - | Referência |  |
| Sim | 644 | 50,1 | 13 | 68,4 | 0,116 | 2,15 (0,83 – 5,56) | 2,85 (1,01 – 8,02) |
| ***Uso de preservativo na última relação com parceiro casual*** |  |  |  |  |  |  |  |
| Não | 210 | 16,3 | 4 | 21,1 | - | Referência |  |
| Sim | 338 | 26,3 | 4 | 21,1 | 0,332 | 0,51 (0,13 – 2,00) |  |
| Não teve parceiro casual nos últimos 6 meses | 713 | 55,5 | 10 | 52,6 | 0,754 | 0,83 (0,26 – 2,63) |  |
| ***Nos últimos 6 meses foi diagnosticada com alguma IST*** |  |  |  |  |  |  |  |
| Não | 1043 | 81,2 | 14 | 73,7 | - | Referência |  |
| Sim | 246 | 18,8 | 5 | 26,3 | 0,443 | 1,49 (0,54 – 4,13) |  |

IC: intervalo de confiança; Anti-HCV: anticorpos totais contra o vírus da hepatite C; IST: Infecção sexualmente transmissível; SM: salário mínimo.

*Renda mensal (sm): R$1.100,00.
