# Supplementary material for: Hepatitis A, B and C prevalence among transgender women and travestis in five Brazilian capitals between 2019-2021
Source: Rev Bras Epidemiol. 2024 Aug 19;27(Suppl 1):e240005.supl.1. doi: 10.1590/1980-549720240005.supl.1 (PMC11338541; doi:10.1590/1980-549720240005.supl.1)
Supplement: Translation

# Translation: Prevalência das hepatites A, B e C entre mulheres trans e travestis em cinco capitais brasileiras entre 2019-2021

**DOI:** 10.1590/1980-549720240005.supl.1.2

**Authors:** Regina Célia Moreira, Maria Amélia de Sousa Mascena Veras, Carolina Amianti, Daniel Jason McCartney, Vanessa Cristina Martins Silva, Marcilio Figueiredo Lemos, Adriana Parise Compri, Elaine Lopes de Oliveira, Katia Cristina Bassichetto, Andréa Fachel Leal, Daniela Ruva Knauth, Laio Magno, Inês Dourado, Lenice Galan, Paula Andrea Morelli Fonseca, Rita Suely Bacuri de Queiroz, Roberto José Carvalho da Silva, Sandra Araujo, Marcia Eiko Miyachi, Claudio de Sousa Soares, Luciana Mitie Kawai Ahagon, Philippe Mayaud, Sandro Sperandei, Ana Rita Coimbra Motta-Castro

**Affiliations:** I Instituto Adolfo Lutz, Centro de Virologia, Laboratório de Hepatites Virais –São Paulo (SP), Brasil.; II Núcleo de Pesquisa e Direitos Humanos em Saúde da População LGBT+ – São Paulo (SP), Brasil.; III Santa Casa de São Paulo, Faculdade de Ciências Médicas – São Paulo (SP), Brasil.; IV Universidade Federal de Mato Grosso do Sul – Campo Grande (MS), Brasil.; V London School of Hygiene & Tropical Medicine, Faculty of Infectious & Tropical Diseases, Department of Clinical Research – London, United Kingdom.; VI Universidade Federal do Rio Grande do Sul – Porto Alegre (RS), Brasil.; VII Universidade do Estado da Bahia, Departamento de Ciências da Vida – Salvador (BA), Brasil.; VIII Universidade Federal da Bahia, Instituto de Saúde Coletiva – Salvador (BA), Brasil.; IX Fiocruz Amazônia, Instituto Leônidas e Maria Deane – Manaus (AM), Brasil.; X Secretaria de Estado da Saúde de São Paulo, Centro de Referência e Treinamento em DST/Aids – São Paulo (SP), Brasil.; XI Laboratório do Centro de Referência e Treinamento DST/Aids – São Paulo (SP), Brasil.; XII Estern Sydney University, Translation Health Research Institute – Penrityh, Australia.; XIII Fundação Oswaldo Cruz – Campo Grande (MS), Brasil.

**Keywords:** Hepatite A, Hepatite B, Hepatite C, Prevalência, Mulheres trans

## Abstract

**Objetivo:**

Estimar as prevalências e fatores associados com as hepatites A, B e C em mulheres trans e travestis em cinco regiões do Brasil.

**Métodos:**

Estudo transversal com mulheres trans e travestis em cinco capitais brasileiras (Campo Grande, Manaus, Porto Alegre, Salvador e São Paulo), entre dezembro/2019 e julho/2021. As amostras foram submetidas à detecção de marcadores das infecções pelos vírus das hepatites A (HAV), B (HBV) e C (HCV), utilizando-se testes rápidos e quimioluminescência. Amostras positivas foram submetidas à detecção de HBV-DNA e HCV-RNA por PCR em tempo real e genotipadas por sequenciamento de Sanger.

**Resultados:**

As análises de 1.317 amostras indicaram taxas de prevalências nas mulheres trans e travestis recrutadas de 69,1%, 24,4% e 1,5% para exposição ao HAV, HBV e HCV, respectivamente. Elevada taxa de suscetibilidade ao HBV (35,7%) e baixa prevalência do marcador vacinal (40,0%) foram observadas. Mostraram-se associadas à presença de anti-HAV: idade maior que 26 anos, autodeclarar-se preta-parda, ter apenas educação básica, história de encarceramento e uso de preservativo na última relação sexual com parceiro casual. Quanto à exposição ao HBV, foi associada a idade maior que 26 anos, cor da pele preto-parda, ter sido profissional do sexo e história de encarceramento. Idade maior de 37 anos, história de abuso sexual e consumo frequente de álcool foram associadas ao HCV.

**Conclusão:**

As maiores prevalências de HAV nessa população encontram-se nas regiões Norte e Nordeste. Com relação ao HBV, a prevalência encontrada foi superior à encontrada na população geral, sugerindo maior vulnerabilidade. A prevalência do HCV foi semelhante à encontrada na população geral.

## INTRODUÇÃO

Em 2016, a Organização Mundial da Saúde (OMS) propôs a todos os países a eliminação das hepatites virais até o ano de 2030, considerando como principais desafios a redução da mortalidade, a queda da incidência, o aumento da oferta do diagnóstico e, principalmente, tornar os tratamentos acessíveis a toda população^1,2^.

Estudos epidemiológicos recentes entre populações-chave (*gays* e os outros homens que fazem sexo com homens (HSH), pessoas trans, pessoas que fazem uso de álcool e outras drogas, pessoas privadas de liberdade e trabalhadores sexuais) indicam que têm sido os grupos mais afetados para infecções pelos vírus da imunodeficiência adquirida (HIV), hepatite B (HBV) e hepatite C (HCV) e, também, para outras infecções sexualmente transmissíveis (IST)^3,4^. A população de mulheres trans e travestis apresentam alto risco para adquirir IST como HIV, sífilis e hepatite B^5,6^.

A saída precoce de casa/escola, discriminação e preconceito vivenciados por muitas delas, principalmente as mais pobres e negras, as levam, muitas vezes, à exclusão social e, consequentemente, à prostituição como estratégia de sobrevivência. Fatores diretamente relacionados à prostituição como práticas sexuais de risco, elevado número de parceiros sexuais, consumo de drogas ilícitas e bebidas alcoólicas, baixo nível educacional, marginalização socioeconômica e violência tornam essa população mais vulnerável em adquirir IST^5-7^.

A infecção pelo HAV está diretamente relacionada às condições de higiene da população e algumas práticas sexuais. Mulheres em situação de rua apresentam alta prevalência, por apresentarem maior probabilidade de contato com água e alimentos contaminados, o que contribui para a ocorrência das infecções de transmissão fecal/oral^8^.

No Brasil, as vacinas contra hepatites A e B estão disponíveis em todo o território nacional. A vacinação contra hepatite B é oferecida desde os anos 2000 e, gradualmente, tornou-se disponível para adultos até 49 anos. A vacina contra hepatite A, inicialmente restrita a pacientes com alguma doença grave, como as pessoas que vivem com HIV, imunossuprimidas ou portadoras crônicas das hepatites B e C^8-10^, passou, desde 2014, a fazer parte do Calendário Nacional de Vacinação, sendo ofertada a todas as crianças a partir dos 12 meses. Em relação ao HCV, a principal estratégia é o diagnóstico precoce da infecção ativa, desde que haja disponibilidade do tratamento eficaz na rede pública com grande possiblidade de cura para a população mais exposta a essa infecção^11-13^.

Diante do exposto, o presente estudo tem por objetivo estimar as prevalências e fatores associados das hepatites A, B e C em mulheres trans e travestis, em cinco regiões do Brasil, assim como identificar os genótipos/subtipos do HBV e HCV circulantes na população estudada.

## MÉTODOS

O presente estudo integra o projeto intitulado "Estudo de Prevalência da Sífilis e Outras Infecções Sexualmente Transmissíveis entre Travestis e Mulheres Transexuais no Brasil: Cuidado e Prevenção", que foi do tipo transversal, realizado em cinco capitais brasileiras (Campo Grande, Manaus, Porto Alegre, Salvador e São Paulo), entre dezembro de 2019 e julho de 2021.

O recrutamento foi realizado por meio de amostragem dirigida por respondedores (*respondent driven sampling* — RDS). Após assinatura do TCLE, todas as participantes, responderam a um questionário sobre dados sociodemográficos e fatores comportamentais, além de antecedentes de vacinação contra hepatite A e B.

Após a entrevista, as participantes foram encaminhadas para a coleta de sangue por punção venosa, para a realização dos testes rápidos (TR) para a pesquisa de anti-HIV-1/2, anti-*Treponema pallidum*, antígeno de superfície HBV (HBsAg) e anti-HCV. Todas as amostras foram encaminhadas para São Paulo, para os Laboratórios de Hepatites Virais do Instituto Adolfo Lutz (IAL) e do Centro de Referência e Treinamento em IST/Aids (CRT) para a realização dos ensaios sorológicos e moleculares. Todas as amostras foram submetidas à detecção de anti-HAV total, anti-HAV IgM, anti-HBc total e anti-HBs pela técnica de imunoensaio de micropartículas quimioluminescente (*chemiluminescent microparticle imunoassay* — CMIA), utilizando-se o sistema Architect^®^ i1000 (Abbott Ireland, Diagnostics Division, Sligo, Ireland), de acordo com as orientações do fabricante.

As amostras reagentes ao HBsAg (TR) e anti-HCV (TR) foram submetidas à detecção e quantificação do HBV-DNA e HCV-RNA, utilizando-se a técnica de PCR em tempo real (qPCR) (Abbott^®^ Real Time, Des Plaines, EUA). As amostras com carga viral detectadas foram genotipadas pela técnica de PCR convencional, seguindo os protocolos descritos por Sitnik et al.^14^ e Gomes-Gouvêa et al.^15^ para o HBV e, para a genotipagem do HCV, foi utilizado o protocolo descrito por Santos et al.^16^ Os materiais genéticos amplificados foram sequenciados pelo método de Sanger et al.^17^, seguindo as orientações da bula do kit do BigDye™ Terminator v3.1 Cycle Sequencing Kit (Applied Biosystems, Foster City, CA, USA) e analisados no Sequenciador Automático ABI3500 (Applied Biosystems, Thermo Fisher Brand, Foster City, CA, USA). Os genótipos foram confirmados, pelo uso da ferramenta de genotipagem Geno2pheno^18^. Maiores detalhes metodológicos do estudo podem ser obtidos em Veras et al.^19^.

Os desfechos para este estudo foram a exposição e a presença de doença ativa dos vírus HAV, HBV e HCV. Para HAV, a exposição (HAV-Expo) foi determinada pela positividade do marcador anti-HAV total. A prevalência de doença ativa (HAV-Prev) foi determinada pelo marcador anti-HAV IgM. Em relação ao HBV, a exposição (HBV-Expo) foi determinada pela positividade para anti-HBc total e(ou) HBsAg, enquanto a prevalência da doença ativa (HBV-Prev) foi detectada pela presença do HBsAg associado ao anti-HBc total. As participantes com positividade isolada para anti-HBs (HBV-Vac), foram consideradas imunes por vacinação contra hepatite B e aquelas sem a presença de nenhum marcador positivo para HBV foram consideradas “suscetíveis” à infecção por HBV (HBV-Susc). A exposição ao HCV (HCV-Expo) foi detectada pela positividade do anti-HCV, enquanto a prevalência ativa da doença (HCV-Prev) foi determinada pela investigação da presença de carga viral HCV-RNA. Com base na literatura, foram incluídas um total de 20 variáveis de exposição possivelmente associadas a cada um dos seis desfechos descritos anteriormente.

Os desfechos principais foram apresentados em frequências absoluta e relativa, com intervalo de confiança de 95% para a amostra total e por cidade/sítio de coleta. As variáveis de exposição foram apresentadas também sob a forma de frequências absoluta e relativa. Modelos logísticos univariados foram utilizados para análise exploratória da associação com cada desfecho. A seleção dos modelos multivariados finais iniciou-se pela seleção das variáveis cujo modelo bivariado resultou em um p-valor igual ou inferior a 0.3^20^ como candidatas para inclusão no modelo. A seleção do modelo final foi realizada por meio da função GLMERSelect, do pacote “StatisticalModels” em R, versão 4.2^21^. A função realiza uma seleção *stepwise* dos efeitos fixos e interações em um modelo linear generalizado (logístico) de efeitos mistos, tendo a cidade/sítio de coleta como interceptos aleatórios. Os resultados das associações foram apresentados como razão de chances pontual (odds ratio — OR) e respectivo intervalo de confiança de 95%.

O projeto foi aprovado pelo Comitê de Ética em Pesquisa (CEP) da Santa Casa de Misericórdia de São Paulo (CAAE 05585518.7.0000.5479 - Nº parecer: 3.126.815 - 30/01/2019), assim como pelas demais instituições participantes.

## RESULTADOS

Participaram do estudo 1.317 mulheres trans e travestis, sendo 403 recrutadas em São Paulo, 192 em Porto Alegre, 201 em Salvador, 181 em Campo Grande e 340 em Manaus. Considerando o total de participantes nas cinco capitais, as idades média e mediana foram, respectivamente, 32,5 (DP 10,0) e 31 anos. A maioria se identificou como mulher transexual (66,8%), solteiras (70,1%) e relatou ter cursado ensino médio completo e incompleto (54,3%). Quanto à ocupação, 24,6% informaram ser autônomas, 21,3% profissional do sexo e 22,5% estavam desempregadas no momento da entrevista.

As Tabelas 1 e 2 mostram as prevalências dos marcadores sorológicos para HAV, HBV e HCV na população estudada. O número final de amostras analisadas variou de acordo com cada hepatite e foi menor do que o número de amostras coletadas, devido ao volume de soro/plasma/sangue total insuficiente para a realização dos testes sorológicos e(ou) moleculares. Um total em 1.270, 1.206 e 1.285 amostras foram analisadas para investigação dos marcadores sorológicos das infecções causadas pelo HAV, HBV e HCV, respectivamente.

**Tabela 1:** Prevalência dos marcadores sorológicos das hepatites A, B e C entre mulheres trans e travestis.

Marcadores sorológicos	n	Positivo	%	IC95%
Hepatite A				
Anti-HAV total	1.270	877	69,1	66,6–71,6
Anti-HAV IgM	1.270	34	2,7	1,8–3,6
Hepatite B				
Exposição ao HBV (anti-HBc e(ou) HBsAg)	1.206	294	24,4	22,0–26,8
Anti-HBs isolado	1.206	482	40,0	37,2–42,8
Ausência de marcador de exposição	1.206	430	35,7	33,0–38,4
Hepatite C				
Anti-HCV	1.285	19	1,5	0,8–2,2

IC: intervalo de confiança, HBV: vírus da hepatite B; HBsAg: antígeno de superfície do HBV; anti-HBs: anticorpo contra o antígeno de superfície do HBV; anti-HBc total: anticorpo contra o antígeno do core do HBV; anti-HAV IgM: anticorpos da classe IgM contra o vírus da hepatite A; anti-HAV total: anticorpos totais contra o vírus da hepatite A; anti-HCV: anticorpos totais contra o vírus da hepatite C.

**Tabela 2:** Prevalência dos marcadores sorológicos das hepatites A, B e C entre mulheres trans e travestis por capital brasileira.

	HAV_Total	HAV_IgM	Anti_HBc T	HBsAg	HBV_Susc	Anti-HBs	HCV_Expo
São Paulo							
P (IC95%)	71,3 (66,9–75,7)	2,7 (0,9–4,5)	28,3 (24,0–33,0)	1,8 (0,2–3,4)	23,8 (19,6–28,0)	47,8 (42,9–52,7)	0,7 (0–2,5)
n/N	286/401	11/401	114/400	7/400	95/400	191/400	3/402
Porto Alegre							
P (IC95%)	57,4 (50,3–64,5)	1,6 (0–5,5)	37,1 (30,0–44,2)	2,7 (0–6,0)	26,3 (19,8–32,8)	36,6 (29,5–43,7)	5,3 (1,6–9,0)
n/N	108/188	3/188	69/186	5/186	49/186	68/186	10/188
Salvador							
P (IC95%)	58,8 (51,5–66,1)	2,8 (0–6,2)	25,0 (17,1–32,9)	0 (---)	38,7 (29,9–47,5)	36,3 (27,6–45,0)	0 (---)
n/N	104/177	5/177	31/124	0/124	48/124	45/124	0/182
Manaus							
P (IC95%)	80,3 (76,0–84,6)	3 (0,9–5,1)	14,8 (10,9–18,7)	1,8 (0–3,7)	48,4 (43,0–53,8)	36,8 (31,6–42,0)	1,2 (0–3,1)
n/N	269/335	10/335	50/337	6/337	163/337	124/337	4/336
Campo Grande							
P (IC95%)	65,1 (57,8–72,4)	3 (0–6,6)	18,9 (12,5–25,3)	1,3 (0–12,7)	47,2 (39,3–55,1)	34 (26,5–41,5)	1,1 (0–11,1)
n/N	110/169	5/169	30/159	2/159	75/159	54/159	2/177

P: positividade; IC: intervalo de confiança; n: total de resultados positivos; N: total analisado; HAV total: anticorpos totais contra o vírus da hepatite A; HAV IgM: anticorpos da classe IgM contra o vírus da hepatite A; Anti-HBc T: anticorpos totais contra o antígeno do core do vírus da hepatite B; HBsAg: antígeno de superfície do vírus da hepatite B; HBV_Susc: ausência de marcador sorológico para o vírus da hepatite B; Anti-HBs: anticorpo contra o antígeno de superfície do vírus da hepatite B; HCV_Expo: anticorpos totais contra o vírus da hepatite C.

No que diz respeito aos marcadores para HAV, 877 participantes (69,1% IC95% 66,6–71,6) apresentaram positividade para o anti-HAV total. A maior prevalência foi encontrada em Manaus (80,3%), seguida por São Paulo (71,3%), Campo Grande (65,1%), Salvador (58,8%) e Porto Alegre (57,4%). A positividade para anti-HAV IgM foi observada em 34 amostras (2,7%; IC95% 1,8–3,6). A prevalência de doença ativa variou entre 3% em Manaus e Campo Grande, seguida de 2,8% em Salvador, 2,7% em São Paulo e 1,6% em Porto Alegre.

Um total de 1.206 amostras foi submetido à detecção de marcadores sorológicos de exposição ao HBV. Dessas, 294 apresentaram positividade para anti-HBc total e(ou) HBsAg, resultando em uma prevalência de 24,4% (IC95% 22,0–26,8). As cidades de São Paulo (28,3%) e Porto Alegre (37,1%) apresentaram as maiores taxas de exposição ao HBV. O HBsAg associado ao anti-HBc total foi detectado em 22 participantes e, em apenas uma, o HBsAg foi encontrado isolado. A presença do HBsAg no TR foi observada em 1,8% (22/1.206; IC95% 1,2–2,7) das amostras analisadas, variando de 2,7% em Porto Alegre a 1,3% em Campo Grande. O anti-HBc total associado ao anti-HBs foi detectado em 24,4%, e a presença do anti-HBc isolado foi encontrado em 3,4% das amostras.

Em 40,0% (482/1.206) das participantes, verificou-se positividade isolada do marcador anti-HBs, sugerindo presença de resposta imune vacinal ao HBV. A maior taxa foi observada em São Paulo (47,8%), seguido de Manaus (36,8%), Porto Alegre (36,6%), Salvador (36,3%) e Campo Grande (34,0%). Por outro lado, em 35,7% (430/1.206), nenhum marcador sorológico para hepatite B foi detectado, sugerindo suscetibilidade à infecção pelo HBV. A maior taxa de suscetibilidade à infecção pelo HBV foi encontrada em Manaus (48,4%), seguida de Campo Grande (47,2%), Salvador (38,7%), Porto Alegre (26,3%) e São Paulo (23,8%).

Entre as 22 amostras que apresentaram positividade ao HBsAg no TR, 19 foram submetidas a detecção de HBV DNA. A presença de HBV DNA foi detectada em 10 amostras e, em apenas três, foi possível a realização da genotipagem. Os genótipos encontrados foram HBV-F (02/03) e HBV-A (01/03) (dados não apresentados).

Marcadores sorológicos de exposição ao HCV (anti-HCV) foram detectados em 1.285 amostras de sangue e dessas, 19 amostras apresentaram positividade ao anti-HCV, resultando em uma prevalência de 1,5% (IC95% 0,8–2,2). As taxas de prevalência de exposição ao HCV variaram de 5,3% (Porto Alegre) a 0,7% (São Paulo). Não foi encontrada nenhuma amostra positiva para o anti-HCV na cidade de Salvador. Manaus e Campo Grande apresentaram taxas de prevalência de anti-HCV de 1,2% e 1,1%, respectivamente (Tabelas 1 e 2).

As 19 amostras positivas para o anti-HCV no TR foram enviadas ao laboratório de hepatites do IAL para pesquisa do HCV-RNA. Em três amostras, não foi possível a realização do ensaio devido ao volume insuficiente de amostra, e oito amostras apresentaram resultado não detectável. A presença de HCV-RNA foi detectada em oito amostras e elas foram submetidas à genotipagem.

O genótipo predominante foi o 3a, detectado em 50,0% (04/08), seguido pelos genótipos 1a, 25,0% (02/08) e 1b, com 25% (02/08) (dados não apresentados).

As Tabelas suplementares 1, 2 e 3 apresentam as análises bivariada e multivariada dos fatores associados às infecções pelo HAV, HBV e HCV na população estudada, respectivamente. Após análise multivariada, as variáveis que permaneceram associadas à presença de anti-HAV total foram: idade maior que 26 anos, autodeclarar-se da cor preta/parda, ter apenas educação básica, história de encarceramento, ter usado preservativo na última relação sexual com parceiro casual*.* Não ter tido relação sexual nos últimos seis meses e ser homossexual, *gay* ou lésbica foram fatores protetores para a positividade ao anti-HAV total. Com relação à infecção pelo HBV, idade maior que 26 anos, autodeclarar-se como preta/parda, ser ou ter sido profissional do sexo, estar em algum relacionamento, moradia instável (em situação de rua ou sem endereço fixo, abrigo ou instituição, pensão/albergue ou casa de prostituição) e história de encarceramento foram associados à presença de marcadores sorológicos de exposição ao HBV na população estudada. História de abuso sexual foi fator protetor para infecção pelo HBV. Após a análise multivariada, verificou-se que idade maior ou igual a 37 anos, história de abuso sexual e consumo de álcool quatro ou mais vezes por semana permaneceram como fatores associados à infecção pelo HCV.

## DISCUSSÃO

Os resultados do presente estudo indicam baixa cobertura vacinal contra as hepatites A e B entre as mulheres trans e travestis estudadas, apesar da disponibilidade desses imunobiológicos no Sistema Único de Saúde (SUS)^22^. Esses resultados foram semelhantes aos encontrados por Choi et al.^23^, em San Francisco, Estados Unidos da América (EUA), e por Rezende^24^ e Oliveira et al.^25^, no Brasil. Tais resultados podem demonstrar a vulnerabilidade da população de mulheres trans e travestis à infecção pelo HBV e ressaltam a necessidade de implantação de estratégias para sensibilizar e aumentar a participação dessa população em programas de vacinação.

As maiores prevalências de hepatite A confirmadas no Brasil encontram-se nas regiões Norte e Nordeste, correspondendo a 55,4% de todos os casos, sendo a maior prevalência no estado do Amazonas (8,35%)^26^. Resultado similar foi encontrado na população estudada, uma vez que Manaus apresentou a maior prevalência das cinco cidades participantes, seguida por São Paulo. A prevalência encontrada em São Paulo pode estar relacionada com o expressivo aumento no número de casos do surto de hepatite A que ocorreu entre HSH e homens *gays* na mesma faixa etária^25^ encontrada no presente estudo. Nessa epidemia, ocorrida entre 2016-2017, foram registradas 155 hospitalizações, quatro casos de hepatite fulminante, sendo que dois evoluíram a óbito^27^.

Destaca-se maior prevalência de anti-HAV total entre mulheres de raça pretas/pardas, resultado semelhante com a associação dessa variável à presença de marcador de infecção do HAV^26^. É importante ressaltar que a população trans/travesti negra inicia a sua vida sexual mais cedo do que as brancas e é quem alega ter sofrido mais violência sexual entre a infância e a adolescência e vivido em situação de rua, fatores que as tornam mais expostas e mais suscetíveis às infecções de transmissão fecal-oral^27,28^. Nossos dados corroboram os dados de Castro et al.^29^, que observaram prevalência em mulheres trans na região Centro-Oeste do Brasil de 69.7%. A população participante desse estudo foi acima de 18 anos e, conforme já descrito na literatura^30^, casos de infecção aguda em adultos podem apresentar quadros clínicos mais graves. A vacinação contra hepatite A na população de mulheres trans e travestis e a utilização de preservativos como prática de sexo seguro durante o sexo oral-anal são algumas das medidas eficazes para prevenção da hepatite A.

Adicionalmente, há um achado que contraria a plausibilidade e a literatura^8,27^. A exposição à violência sexual aparece como fator protetor em nosso estudo, assim como a associação da hepatite A com a utilização de preservativo na ultima relação. Essas associações podem estar sendo confundidas pela presença de fatores não mensurados.

Com relação à hepatite B, a prevalência global para os marcadores de exposição ao HBV encontrada neste estudo, nas mulheres trans e travestis estudadas, foi superior à encontrada no inquérito de base populacional, realizado entre os anos de 2005 e 2009, nas 26 capitais e no Distrito Federal (7,4%; IC95% 6,8–8,0), sugerindo maior vulnerabilidade dessa população à infecção pelo HBV^31^. Entretanto foi similar ao encontrado no Centro-Oeste do Brasil em 2018^24^ e inferior às prevalências encontradas em outros países latino-americanos como a Argentina^32^, com 40,2%, e o Uruguai^6^, com 50,5%.

A positividade para o HBsAg observada nas mulheres trans e travestis estudadas foi superior à prevalência de HBsAg encontrada no estudo de base populacional realizado nas capitais brasileiras^30^. Foi semelhante também à encontrada na Argentina (1,9%)^32^ e em Campo Grande-MS (2,7%)^24^ em pessoas trans e profissionais do sexo. Contudo foi inferior ao índice encontrado em países como o Paquistão^33^ (3,4%) e o Uruguai^6^ (3,0%).

Após análise multivariada, idade acima de 26 anos foi independentemente associada à infecção pelo HBV. Provavelmente, devido ao aumento do risco de exposição ao vírus ao longo da vida, principalmente pela via sexual essa associação tem sido amplamente relatada^34,35^. Nesse contexto, ser ou ter sido profissional do sexo também foram fatores associados à hepatite B nessa população, semelhante ao encontrado em outros estudos^35-37^. Cabe destacar que a maioria da população estudada já trabalhou ou trabalha como profissionais do sexo, sendo a taxa observada, semelhante à encontrada em outros estudos^7,24,38^. Estudos têm mostrado que a necessidade financeira, o estigma social, a dificuldade de acesso ao mercado de trabalho e a marginalização a saúde são os principais motivos para essa população ingressar no trabalho como profissional do sexo^39,40^. Fatores diretamente ligados à prostituição, como o elevado número de parceiros e práticas sexuais de risco, torna essa população mais vulnerável para adquirir IST, como o HBV^34,38,41^.

Fatores socioeconômicos, como o fato de viver em moradias instáveis, estão relacionados com a exposição ao HBV. Estudos realizados no Brasil^7,24,38^ e na Índia^41^ também verificaram baixo nível socioeconômico no grupo das mulheres trans. O nível socioeconômico está diretamente relacionado com as condições precárias de moradia, como baixas condições higiênico-sanitárias e violência, aumentando, assim, o risco para infeções.

A hepatite C assume importância na população de mulheres trans, principalmente, pela transmissão através de uso de drogas injetáveis e(ou) inaláveis; pessoas privadas de liberdade; e pessoas infectadas pelo HIV^12^. A maior taxa de detecção para HCV foi encontrada na capital do Rio Grande do Sul, que é o estado com maior taxa da infecção no país^26^. No entanto, de forma geral, a prevalência da HCV na população estudada foi semelhante à encontrada na população geral, que, de acordo com o inquérito nacional, é de 1,38%^31^.

A prevalência do HCV no grupo estudado difere significativamente daquela encontrada nos EUA. Dois estudos do tipo RDS realizados com mulheres trans no estado da Califórnia nos anos de 2017 e 2020 encontraram prevalências de 23,8 e 23,9%, respectivamente^42,43^. A associação da idade como fator de risco para HCV, já descrita na literatura^32^, aparece tanto no contexto brasileiro quanto no estadunidense.

A associação da infecção pelo HCV com o consumo de álcool e história de abuso sexual pode ser explicada pelo fato de que a violência contra a mulher ou criança gera consequências de diferentes aspectos para a vítima, podendo trazer impactos psicológicos e comportamentais, com maiores chances de se envolverem futuramente com o uso de álcool e(ou) outras drogas^44,45^.

Algumas limitações foram observadas no presente estudo. Por se tratar de um estudo transversal, não se pode estabelecer uma relação de causalidade entre as variáveis de exposição analisadas e os desfechos. As taxas de suscetibilidade e da presença de marcadores de resposta vacinal contra hepatite B podem ter sido subestimadas, uma vez que os níveis de anti-HBs diminuem ao longo do tempo e podem atingir níveis indetectáveis. Autorrelato e viés de memória são também algumas das limitações encontradas. Além disso, o incentivo financeiro oferecido pelo método de recrutamento pode ter atraído mais mulheres trans e travestis de baixa renda para participar do estudo.

A OMS reconhece que algumas populações-chave, como a de pessoas trans, apresentam taxas de prevalência de hepatites virais superiores às encontradas na população em geral. Conhecer os aspectos epidemiológicos e moleculares das hepatites A, B e C nesses grupos populacionais, como o grupo de mulheres trans e travestis, é importante para auxiliar no direcionamento das políticas públicas de saúde dirigidas a essa população^1^. Nesse sentido, os dados apresentados evidenciam o impacto das hepatites virais nesse grupo populacional, que apresenta características de alta vulnerabilidade, refletida na irregularidade de adoção de práticas sexuais seguras, histórico de IST, estigma social, violência e dificuldade de acesso aos serviços de saúde. Os resultados indicam a necessidade de políticas públicas direcionadas especificamente para esta população-chave com intuito de aumentar a cobertura vacinal e o acesso à testagem e ao tratamento.
